# Ownership and utilization of long-lasting insecticide-treated bed nets in Afar, northeast Ethiopia: a cross-sectional study

**Published:** 2012-12-26

**Authors:** Kassahun Negash, Berhane Haileselassie, Awoke Tasew, Yesuf Ahmed, Medhanit Getachew

**Affiliations:** 1 African Medical and Research Foundation Ethiopia, AMREF Ethiopia; 2 Malaria Control and Evaluation Partnership in Africa (MACEPA), Ethiopia

**Keywords:** Long-lasting insecticide-treated nets, determinants, utilization, Ethiopia

## Abstract

**Introduction:**

Malaria is the leading cause of morbidity and mortality in Afar Region. Distribution of Long Lasting Insecticide Treated Bed Nets (LLINs) has been one of the major interventions to combat malaria. However, ownership and utilization of these nets are not well known.

**Methods:**

A community based cross-sectional study was conducted using interviewer-administered questionnaires to study LLIN coverage. After systematic random sampling of the study population, data on utilization of LLINs and factors influencing this utilization were collected. Analysis of these data was done using SPSS software.

**Results:**

Household possession of at least one LLIN in the surveyed households was found in 648(86.1%) households. Ownership of at least two nets was found among 419(55.6%) surveyed households. The proportion of children under 5 years of age who slept under treated nets during the night preceding the survey was 728(82.0%) and 676 (76.1%) in the surveyed households for reported and observed respectively. Likewise, the proportion of pregnant women who slept under treated nets was 166 (79.1%) and 147(70.0%) for reported and observed respectively. Among the potential determinants explored regarding utilization of LLINs, age, occupation, and radio possession were found to be significantly associated with LLIN utilization. Households that did not possess radio were 0.38 times (95%CI= 0.25-0.59) less likely to let their children under five and pregnant women sleep under LLIN.

**Conclusion:**

The LLINs coverage and utilization among the pastoralist community are promising. Strengthening of the primary health care unit and timely replacement of LLINs are critical for improved outcomes.

## Introduction

There were an estimated 247 million cases of malaria and 3.3 billion people at risk of the disease in 2006, causing nearly a million deaths, mostly of children under 5 years [1]. In Ethiopia, almost 75% of the country is malarious and an estimated 51 million (68%) of the population lives in areas at risk of malaria [2]. Malaria was the leading cause of health problems in the country. In 2005/6, the disease was reported as the first cause of illness and death in outpatient visits [2].

Malaria transmission in Ethiopia is unstable and characterized by frequent and often large-scale epidemics [1, 3]. Apart from being a major public health problem, the disease has also been identified as a potential impediment to the development of water harvesting, irrigation for agriculture and settlement in fertile underutilized low land areas with potential to enable the country achieve food security and improved household income. In 2006/7 the disease was reported as the sixth cause of illness for all outpatient visits and the seventh cause of illness for female outpatient visits [4].

Malaria is the leading cause of morbidity and mortality in Afar Region. Malaria transmission in the region is generally unstable, with perennial transmission in areas along the Awash River Valley where modern irrigation schemes such as Sabure, Amibara, Bure-Mudaytu, Gewane, Tendaho and Asayta extensively take place along the shores of Awash River. Consequently, the economic impact of malaria is far greater than for any other communicable disease. There were several instances of epidemics following flooding of Awash River; the worst incident was that of the 2000 that led to serious eruption of malaria and other water-borne disease epidemics [2].

Almost all parts of the region are malarious. As in the rest of Ethiopia, malaria is highly seasonal with great variation from year to year, and generally leaving the population with little protective immunity. Plasmodium falciparum and Plasmodium Vivax are the two dominant malaria parasites prevailing in the region.

Due to an increasing burden of malaria, a range of effective malaria control interventions are currently underway in Ethiopia through the support of Roll Back Malaria (RBM) and Global Fund to fight AIDS, Tuberculosis and Malaria (GFATM) to improve access and equity to preventive as well as curative health services [3, 4]. These initiatives include prompt and effective treatment of malaria, selective vector control including insecticide treated mosquito nets (LLINs) and indoor residual house spraying (IRS), and prevention and control of epidemics. Strong IEC/BCC interventions are the core components of each malaria control strategy. Important steps have been taken particularly to scale-up the implementation of LLINs/LLINs in Ethiopia. In 2006/2007, about 16 million LLINs were distributed to about 8 million households in malarious areas of the country [5, 6]. To date, about 20 million LLINs/LLINs have been distributed through Regional Health Bureaus and other stakeholders.

However, the scaling up and effectiveness of malaria control interventions is dependent on the perceptions and household behavioural practices of the local community. Changes in the health care delivery system might not necessarily be followed by changes in behaviour or knowledge about disease causation and prevention among the population. The local socio-cultural context, social and economic factors coupled with poor health service coverage may lead to late treatment seeking behaviour and under utilization of nets [7].

In the study area conditions conducive to both the vector and extrinsic parasite development occur from September through November, following the main rains which start in May and decline in September [8]. Today, there are growing interests in using Long-lasting Insecticide-treated Nets (LLIN) as one of the leading strategies for prevention of malaria [9, 10]. Different trials have shown a promising result that LLIN or curtains reduce all-causes of childhood mortality by 14-33% in rural sub-Saharan Africa [5]. Organizations like UNICEF, WHO, the GFATM and others deliver large amount of LLINs to prevent malaria. Therefore, this study attempted to elucidate whether the delivered LLINs are being possessed and properly utilized particularly by children under five and pregnant women; and identify the factors that affect the possession and utilization of LLINs for malaria prevention in order to achieve the national targets.

## Methods

### Study setting

The study was conducted in six districts, three each for zone three and zone five, in Afar National Regional State, North-eastern Ethiopia. Malaria is the leading cause of morbidity and mortality in the districts. Majority of the population in these districts are semi-pastoralist or pastoralist. The randomly selected sub-districts (kebeles) of these districts were the study area.

### Study population

Randomly selected heads of households residing in the selected districts of zone 3 and 5 during the study period were the study population. In the absence of the household head the wife or any adult household member able to provide information and aged 15 years and above was targeted. People residing temporarily for seasonal work like migrant labourers and visitors were excluded from the study.

### Study design

A community based cross-sectional design was conducted using an interviewer-administered questionnaire to study the coverage of LLINs and prompt treatment of fever. Checklists for early morning survey on direct observation of the LLINs condition were also used to observe the actual behaviour of the community towards LLINs.

### Sample size determination

The required sample size was calculated using EPI INFO software package based on the following assumptions: sample size (n) was determined based on the single proportion for cross-sectional survey assumed; 50% of the population would have observed LLINs utilization; and 5% margin of error at 95% percent confidence level. Thus, a sample of was found 384 was arrived at. However, with a design effect of 2 and non response rate of 5% the total sample size was 806.

### Sampling procedure

Targeted districts and kebeles were first selected using a lottery method from the list of districts and their respective kebeles. From the list, the required households were selected using systematic random sampling where every third unit where households within the Kebeles were distributed based on proportionate population to size.

### Data collection

A community-based cross-sectional study design was employed, using an interviewer-administered structured questionnaire as well as an observation checklist. The questionnaire included questions about the respondents′ socio-demographic characteristics, knowledge and attitudes about malaria and malaria prevention, and possession and utilization of LLINs. To ensure accuracy, the questionnaire was originally prepared in English, translated to Amharic, and then translated back to English. Local field guides translated Amharic questions into Afarigna and responses from Afarigna into Amharic. Checklists for direct observation of LLIN ownership and utilization were used to verify the respondents′ behaviors.

Data in the field were collected by 18 trained data collectors working with 18 field guides/translators, overseen by two supervisors. Before commencing data collection, the principal investigator conducted a two-day workshop to train all data collectors in the overall study goal and objectives as well as how to conduct the questionnaire and observation. The two field supervisors checked all questionnaires on a daily basis. After verifying consistency and completeness, the supervisors submitted the filled questionnaires to the principal investigator. Incorrectly filled questionnaires and those missing responses were sent back to the respective data collector for correction. The principal investigator again rechecked the completed questionnaires to maintain the quality of data. The principal investigator and supervisors also rechecked five percent of the samples in order to crosscheck the collected data.

### Data quality assurance

Questionnaires were checked for completeness on a daily basis by immediate supervisors. After checking for consistency and completeness, the supervisors submitted the filled questionnaire to the principal investigator. Incorrectly filled or missed ones were sent back to respective data collectors for correction. The principal investigator again rechecked the completed questionnaires to maintain the quality of data. The principal investigator and supervisors also rechecked five percent of the samples in order to crosscheck the collected data.

### Data analysis

Data were entered, cleaned and analyzed using SPSS 11.0 statistical package for Windows. Five percent of the data was re-entered in order to compare and assure the quality of the data. In addition to descriptive analyses, crude odds ratios were conducted to determine if significant associations existed between socio-demographic characteristics and LLIN possession and utilization. Logistic regression was used in order to identify predictive variables.

### Ethical considerations

Permission was obtained from the relevant District Administrations and District Health Offices. Data collectors obtained informed consent from all respondents before administering the questionnaire and observation checklist. Permission and ethical issues are different.

## Results

### Socio-demographic characteristics

Although the response rate was 100%, 6.6% of the collected data were excluded from the analysis due to poor data quality. The final sample size was 753 respondents. As shown in Table 1, 52.6% of the respondents were male, although a majority of Zone 3 respondents were female (51.2%). The median age was 33 ± 12.9 years, with a range of 15 to 88 years. Majority (89.5%) of respondents in both zones was from rural areas, and 64.4% were illiterate. Most (76.9%) participants were members of the Afar ethnic group, with a higher (88%) proportion in Zone five. Overall, 90% were Muslims, with a higher proportion (99.5%) in zone five. About 43% of respondents reported that they were pastoralists, although the proportion was higher (52.4%) in Zone Five. Most respondents (79%) were currently married, with the rest single, divorced, or widowed. Of the married respondents, a minority (14.9%) reported being in a polygamous relationship. Household radio possession was higher in Zone Three (51.4%) than in Zone Five (37.8%).

**Table 1:** Socio demographic characteristics of respondents in zone 3 and 5, Afar October 2008

	Respondents by Zone, Number n (%)		
	Zone 3	Zone 5	Total
**Sex**			
Male	159(48.62)	237(55.63)	396(52.59)
Female	168(51.38)	189(44.37)	357(47.41)
**Age in years**			
15-30	136(41.59)	198(46.48)	334(44.36)
31-45	138(42.20)	151(35.45)	289(38.38)
46-60	43(13.15)	60(14.08)	103(13.68)
61 and above	10(3.06)	17(3.99)	27(3.59)
**Residence**			
Urban	27(8.26)	52(12.21)	79(10.49)
Rural	300(91.74)	374(87.79)	674(89.51)
**Education Level**			
Illiterate	206(63.00)	279(65.49)	485(64.41)
Literate	121(37.00)	147(34.51)	268(35.59)
**Ethnicity**			
Afar	204(62.39)	375(88.03)	579(76.89)
Non afar	123(37.61)	51(11.97)	174(23.11)
**Religion**			
Islam	252(77.1)	424(99.5)	676(89.8)
Christian	75(22.9)	2(0.5)	77(10.2)
**Occupational status**			
Government employee	44(13.46)	88(20.66)	132(17.53)
Farmer	31(9.48)	24(5.63)	55(7.30)
Merchant	33(10.09)	34(7.98)	67(8.90)
Daily laborer	94(28.75)	11(2.58)	105(13.94)
Pastoralist	97(29.66)	223(52.35)	320(42.50)
Unemployed	28(8.56)	46(10.80)	74(9.83)
**Marital Status**			
Married monogamy	226(69.11)	257(60.33)	483(64.14)
Married polygamy	39(11.93)	73(17.14)	112(14.87)
Single	23(7.03)	41(9.62)	64(8.50)
Divorced/separated	15(4.59)	33(7.75)	48(6.37)
Widowed	24(7.34)	22(5.16)	46(6.11)
**Possession of radio**			
Yes	168(51.38)	161(37.79)	329(43.69)
No	159(48.62)	265(62.21)	424(56.31)

The average number of members per household was 5.0 ± 2.5 overall. Zone Three had a higher number of average members per household than Zone Five, 5.4 ± 2.7 versus 4.7 ± 2.3. Zone Three households had fewer children under five than Zone Five, however, at 394 and 494, respectively. Zone Three households also reported fewer pregnant women than Zone Five, at 54 and 156, respectively. The mean number of rooms per house was 1.5 in Zone Three and 0.9 in Zone Five. Zone Three respondents had a larger average number of bed or sleeping places (both indoors and outdoors) than Zone Five, at 2.5 ± 1.1 and 2.1 ± 1.3, respectively. The mean number of nets reported per household in Zone Three was 2.3 ± 1.1, while in Zone Five it was 1.7 ± 0.8. The mean number of children and pregnant women per LLIN was 0.9:1, while the overall ratio was 3.0:1.

### Knowledge and practice about malaria and mosquitoes

As shown in Table 2, 95.5% of respondents, including a higher proportion (98.5%) in Zone Three than in Zone Five (93.2%), reported that they had heard of malaria. Furthermore, 89.5% of participants, including a higher percentage in Zone Five (91.8%) than in Zone Three (86.5%), knew that mosquitoes play a role in transmitting the disease. About 82% of respondents correctly identified fever as a symptom of malaria. Other symptoms identified by at least half of the respondents included feeling cold, headache, and vomiting. Most (60.2%) participants in both zones identified children under five years of age as the group most affected by malaria, and 27.9% identified pregnant women as the most affected group.

**Table 2:** Knowledge, attitude and practice of respondents on malaria interventions in Zone 3 and 5, Afar October 2008

	Respondents by zone Number n (%)		
	Zone 3	Zone 5	Total
**Heard of malaria**	322(98.5)	397(93.2)	719(95.5)
**Mosquito bite can be prevented**	299(91.4)	379(89.0)	678(90.0)
**Mosquito could transmit malaria**	283(86.5)	391(91.8)	674(89.5)
**Symptoms of malaria**			
**Fever**	239(73.1)	375(88.0)	614(81.5)
**Feeling cold**	169(51.7)	297(69.7)	466(61.9)
**Headache**	241(73.7)	220(51.6)	461(61.2)
**Vomiting**	165(50.5)	216(50.7)	381(50.6)
**Group most affected by malaria**			
**Children under five**	178(54.4)	275(64.6)	453(60.2)
**Pregnant women**	71(21.7)	139(32.6)	210(27.9)
**Sleeping pattern of children under five**			
**With mother**	156(47.7)	147(34.5)	303(40.2)
**With both parents**	42(12.8)	126(29.6)	168(22.3)
**LLIN ownership:**			
**Yes**	296(90.5)	352(82.6)	648(86.1)
**No**	31(9.5)	74(17.4)	105(13.9)
**Number of LLINs owned**			
**0**	31(9.5)	74(17.4)	105(13.9)
**1**	296(90.5)	352(82.6)	648(86.1)
**2**	233(71.3)	186(43.7)	419(55.6)
**3 or more**	81(24.4)	47(11.0)	128(17.0)
**Reported utilization of LLINs**			
**Children under five**	330(83.8)	398(80.6)	728(82.0)
**Pregnant women**	41(75.9)	125(80.1)	166(79.1))
**Observed utilization of LLINs**			
**Children under five**	312(79.2)	364(73.7)	676(76.1)
**Pregnant women**	40(74.1)	107(68.6)	147(70.0)
**LLIN hanged**			
**Yes**	281(85.9)	340(79.8)	621(82.5)
**No**	46(14.1)	86(20.2)	132(17.5)
**LLIN tucked**			
**Yes**	259(79.2)	282(66.2)	541(71.9)
**No**	68(20.8)	144(33.8)	212(28.1)

### Possession and utilization of LLIN possession

As shown in Table 2, 86.1% of respondents reported owning at least one LLIN. Seventeen percent of participants in both zones owned at least three LLINs. According to respondents, the majority of children under five (82.0%) and pregnant women (79.1%) had slept under an LLIN the night preceding the survey. These reports were largely confirmed through early morning observations by the data collectors, although the observed rates of usage were lower than those reported. During the observations, 82.5% of the surveyed households had at least one LLIN hanging, and 71.9% had tucked the LLIN(s) under a mattress or other sleeping material.

### Determinants of LLIN ownership and utilization

Several variables were found to be significantly linked to LLIN possession when adjusted, as shown in Table 3. Household heads employed as day laborers were 15.9 times more likely than those in other professions to report owning at least one LLIN, while households headed by individuals from non-Afar ethnic groups were 3.3 times more likely than those headed by Afars to own an LLIN. Radio possession was inversely related to LLIN ownership-- those who did not own a radio were 0.2 times less likely than those who did to own at least one LLIN. Table 4 shows determinants of LLIN utilization among the surveyed households. Among the potential determinants regarding utilization of LLINs, several were found to be significantly associated with LLIN utilization, as shown in Table 3. Household leads by people above 60 years were 7.7 times more likely to let their children under five and pregnant women sleep under a net. Pregnant women and children under five living in households headed by a day labourer were 4.8 times more likely than those living in households where the head had another profession to sleep under an LLIN. Finally, household radio possession was again inversely related to LLIN possession. Children under five and pregnant women in households without a radio were 0.4 times less likely to sleep under an LLIN than those from households with a radio.

**Table 3:** Predictors of LLIN utilization for households in zone 3 and 5 Afar, October 2008.

Description		No. (%)	OR, 95% CI (Crude)	OR, 95% CI (Adjusted)
**Age in years**	**15-30**	334(44.36)	1.00	1.00
	**31-45**	289(38.38)	0.82(0.85,1.23)	0.84(0.54, 1.29)
	**46-60**	103(13.68)	1.31(0.70, 2.46)	1.52(0.78, 2.95)
	**Above 60**	27(3.59)	5.32(0.71, 39.73)	7.66(1.01, 58.14)*
**Sex**	**Male**	396(52.59)	1.00	1.00
	**Female**	357(47.41)	1.10(0.76, 1.60)	1.22(0.81, 1.83)
**Occupation**	**Gov. Employee**	132(17.53)	1.00	1.00
	**Farmer**	55(7.3)	1.82(0.70, 4.72)	1.82(0.68, 4.88)
	**Merchant**	67(8.9)	0.84(0.40, 1.76)	0.80(0.35, 1.85)
	**Daily laborer**	105(13.94)	3.67(1.44, 9.33)*	4.08(1.51, 11.03)*
	**Pastoralist**	320(42.50)	0.78(0.47, 1.30)	0.98(0.54, 1.78)
	**Unemployed**	24(9.83)	1.61(0.70, 3.66)	1.58(0.67, 3.70)
**Education**	**Illiterate**	485(64.41)	1.00	1.00
	**Literate**	268(35.59)	1.55(1.02, 2.35)*	1.44(0.87, 2.36)
**Ethnic**	**Afar**	579(76.89)	1.00	1.00
	**Non Afar**	174(23.11)	1.69(1.02, 2.79)*	1.06(0.53, 2.11)
**Radio possession**	**Yes**	329(43.69)	1.00	1.00
	**No**	424(56.31)	0.38(0.25, 0.58)*	0.38(0.25, 0.59)*

* Statistically significant at p<0.05

**Table 4:** Predictors of LLIN possession for households in zone 3 and 5 Afar, October 2008.

Description		No. (%)	OR, 95% CI (Crude)	OR, 95% CI (Adjusted)
**Age in years**	**15-30**	334(44.36)	1.00	1.00
	**31-45**	289(38.38)	0.80(0.55,1.25)	0.83(0.51, 1.34)
	**46-60**	103(13.68)	1.18(0.60, 2.33)	1.40(0.68, 2.89)
	**Above 60**	27(3.59)	4.02(0.54, 30.20)	6.63(0.86, 51.18)
**Sex**	**Male**	396(52.59)	1.00	1.00
	**Female**	357(47.41)	1.30(0.85, 1.97)	1.37(0.87, 2.16)
**Occupation**	**Gov. Employee**	132(17.53)	1.00	1.00
	**Farmer**	55(7.3)	1.79(0.63, 5.03)	1.99(0.68, 5.86)
	**Merchant**	67(8.9)	1.15(0.49, 2.69)	0.61(0.22, 1.69)
	**Daily laborer**	105(13.94)	18.57(2.45, 140.75)*	15.94(2.02, 125.99)*
	**Pastoralist**	320(42.50)	0.74(0.43, 1.29)	1.08(0.57, 2.05)
	**Unemployed**	24(9.83)	1.47(0.61, 3.53)	1.46(0.58, 3.66)
**Religion**	**Islam**	676(89.8)	1.00	1.00
	**Christian**	77(10.2)	4.36(1.35, 14.06)*	0.75(0.17, 3.29)
**Education**	**Illiterate**	485(64.41)	1.00	1.00
	**Literate**	268(35.59)	1.62(1.02, 2.57)*	1.30(0.74, 2.27)
**Ethnic**	**Afar**	579(76.89)	1.00	1.00
	**Non Afar**	174(23.11)	4.17(1.99, 8.74)*	3.30(1.10, 10.07)*
**Radio possession**	**Yes**	329(43.69)	1.00	1.00
	**No**	424(56.31)	0.24(0.14, 0.41)*	0.24(0.14, 0.42)*

* Statistically significant at p<0.05

## Discussion

This study provides, probably for the first time, data on determinants of utilization of LLINs among people in the Afar region of Ethiopia. This is an area where malaria is known to be endemic [1]. Knowledge of possession of these nets and its determinants are important guides for decision-making in malaria control programmes and ultimate control of devastating effects of the disease on the population.

In this study, malaria as a serious communicable disease was better recognized in Zone Three than Zone Five. However, the mode of transmission of malaria through mosquito bite was more mentioned in zone five. This may be explained by the fact that malaria intervention programs have more effect in zone five. This may have resulted in increased mosquito control in Zone Three.

The study showed that the utilization of bed net in the study area by children and pregnant women was 82% and 79% respectively. However, in the direct observation it was observed in 76.1% and 70% children and pregnant women. This finding was consistent with a study conducted in Wonago in Ethiopia where the utilization was 68.8% [5]. Nevertheless, there is discrepancy in the reported and the observed utilization of bed nets by the community. It is clear that the community may be over reporting in view of the fact that the support from Regional Health Bureau may be continued if results are desirable. Thus, the questionnaire based survey may be mostly over reported than the observed one. On the other hand half of the bed nets in the community were tucked under the mattress, and other places where other insects were. In rural Ethiopia where the hygiene problem is prevalent, this problem is given special emphasis than mosquitoes particularly in non malaria transmission season [6, 7].

Even though the majority of households have one LLIN, the utilization was found to be promising. Utilization exceeded the 60% target set in the Roll Back Malaria framework for 2005 [4, 9]. The study identified a high utilization among daily laborer than other community members. This can be explained by the fact that daily laborers were likely to sleep in an area of poor infrastructure where using the LLIN was mandatory to prevent mosquito bite as was the case in a study conducted in a different region of Ethiopia [10]. In addition to that these people were living in a relatively more urbanized town area where there was a possibility to get information through media like radio.

## Conclusion

In the study area the LLINs coverage, utilization and health seeking behavior of the pastoralist community have shown a promising result. There is no significant difference between observed and reported LLINs utilization in the study. Radio message was found to be the most important tool to convey message in the prevention and control of malaria in the study area. More in-depth studies of LLIN utilization should be conducted covering the whole region. Possession of at least two LLINs was found to be low and therefore more LLINs should be distributed in the study community. Locally tailored malaria messages should be developed and transmitted through the local language by radio to improve LLIN utilization. Moreover, better supervision of existing malaria treatment guidelines in formal health facilities and strengthening community based awareness creation activities are very critical.
